# Flavonoids Affect Host-Microbiota Crosstalk through TLR Modulation

**DOI:** 10.3390/antiox3040649

**Published:** 2014-10-17

**Authors:** Francisco J. Pérez-Cano, Malen Massot-Cladera, Maria J. Rodríguez-Lagunas, Margarida Castell

**Affiliations:** 1 Departament de Fisiologia, Facultat de Farmàcia, Universitat de Barcelona; Av. Joan XXIII s/n, Edifici B, 3ª planta, E-08028 Barcelona, Spain; E-Mails: malen.massot@ub.edu (M.M.-C.); margaridacastell@ub.edu (M.C.); 2 Institut de Recerca en Nutrició i Seguretat Alimentària (INSA-UB), E-08028 Barcelona, Spain; E-Mail: mjrodriguez@ub.edu; 3 Departament de Ciències Fisiològiques II, Facultat de Medicina, Universitat de Barcelona, Feixa Llarga s/n, L′Hospitalet de Llobregat, E-08907 Barcelona, Spain

**Keywords:** TLR, polyphenols, inflammation, vegetables, intracellular signaling

## Abstract

Interaction between host cells and microbes is known as crosstalk. Among other mechanisms, this takes place when certain molecules of the micro-organisms are recognized by the toll-like receptors (TLRs) in the body cells, mainly in the intestinal epithelial cells and in the immune cells. TLRs belong to the pattern-recognition receptors and represent the first line of defense against pathogens, playing a pivotal role in both innate and adaptive immunity. Dysregulation in the activity of such receptors can lead to the development of chronic and severe inflammation as well as immunological disorders. Among components present in the diet, flavonoids have been suggested as antioxidant dietary factors able to modulate TLR-mediated signaling pathways. This review focuses on the molecular targets involved in the modulatory action of flavonoids on TLR-mediated signaling pathways, providing an overview of the mechanisms involved in such action. Particular flavonoids have been able to modify the composition of the microbiota, to modulate TLR gene and protein expression, and to regulate the downstream signaling molecules involved in the TLR pathway. These synergistic mechanisms suggest the role of some flavonoids in the preventive effect on certain chronic diseases.

## 1. Host-Microbiota Crosstalk and Dietary Factors

### 1.1. Interaction between Microbiota and the Host

The intestinal mucosa is constantly interacting with a high load of antigens, which comprises, besides those from food, those derived from microbes, both commensal microbiota and invading pathogens. For this reason, the gastrointestinal tract contains the intestinal epithelial cells (IEC) that act as a physical barrier, and the largest part of the immune system: the gut-associated lymphoid tissue (GALT), with approximately 70% of the body immune cells [1]. The main function of the IEC and immune cells located in the intestine is to discriminate precisely between the potential harmful antigens, and then mounting an effective immune response, and those which are innocuous and therefore do not require counteraction from the host, a process known as oral tolerance [2]. The key and ineludible first action in this discrimination consists of the recognition of microbial components in the intestine.

The microbial interaction with host cells (both immune and non-immune cells) is known as crosstalk. Certain microbiota components can be recognized through the pattern-recognition receptors (PRRs). These are host innate immune receptors involved in the detection of pathogens that recognize conserved molecular structures known as pathogen-associated molecular patterns (PAMPs) as well as microbe-associated molecular patterns (MAMPs) (host-commensal interactions) and induce the production of innate effector molecules. These can be located on the cell membrane or in the cytosol joining to bacterial-surface-exposed structures or nucleic acids, respectively. The cytosolic receptors are composed of two major families: the nucleotide oligomerization domain (NOD)—like receptors (NLRs) such as NOD1, NOD2 and NLRP1, and the retinoic acid inducible gene I (RIG-1)—like receptors (RLRs) which recognizes viral RNAs. The cell membranes’ PRRs include toll-like receptors (TLRs) that may reside outside the cell or in specialized vesicular compartments (endosomes) where they detect DNA and RNA derived from viruses and bacteria [3].

PRRs represent the first line of host defense against pathogens, play a pivotal role in both innate and adaptive immunity and can lead to the subsequent inflammatory process. Their activation results not only in local mucosal immunity but also in systemic immune responses. In this sense, small amounts of MAMPS and exogenous antigens from microbes are handled in a homeostatic manner when the PRRs are operating in a healthy manner, leading then to an appropriate pathway activation and balanced mediators secretion. However, dysregulation in the activity of such receptors can lead to the development of chronic and severe inflammation as well as immunological disorders, including septic shock, atherosclerosis, diabetes and cancer [4,5].

It can be of importance to study the influence of diet in such initial activation. Among components present in the diet, polyphenols, mainly flavonoids, have been suggested as dietary factors able to modulate TLR-mediated signaling pathways [5].

### 1.2. Toll-Like Receptors

Each host TLR recognizes distinct PAMPs derived from various microbial pathogens including viruses, bacteria, protozoa and fungi. However, besides their microbial-sensing activity, TLRs also play a role in the recognition of endogenous intracellular molecules released by activated or necrotic cells and extracellular matrix, called damage-associated molecular patterns (DAMPs), which also lead to an inflammatory response which is needed to promote tissue repair [6].

In addition, TLR activation induces the expression of many immune and inflammatory genes via the stimulation of nuclear factor κB (NF-κB) and mitogen-activated protein kinases (MAPKs), such as extracellular signal-regulated kinase (ERK), p38 and c-jun N-terminal kinase (JNK) [7]. Generally, NF-κB and MAPKs, involving p38MAPK or JNK, contribute in activating some proinflammatory cytokines [8,9], whereas activation of ERK promotes cell survival [10].

There are 11 human TLRs identified so far, divided into three groups according to the target in which they interact: lipids and lipopeptides (TLR1, 2, 4 and 6), proteins (TLR5) and nucleic acids (TLR3, 7, 8 and 9). TLR1 forms heterodimers with TLR2 (TLR1/2) and recognizes triacyl lipopeptides. TLR2 in concert with TLR1 or TLR6 recognizes a wide variety of PAMPs, including peptidoglycan, lipopeptides and lipoproteins of Gram-negative bacteria, mycoplasma lipopeptides and fungal zymosan. TLR6 in association with TLR2 (TLR2/6) recognizes diacyl lipopeptides. TLR4 interacts with bacterial lipopolysaccharide (LPS). TLR5 recognizes bacterial flagellin. TLR3 recognizes double-strand RNA, and TLR7 and TLR8 interact with the single-strand RNA found during viral replication. TLR9 recognizes unmethylated deoxycytidyl-phosphate-deoxyguanosine (CpG) motifs commonly present in bacterial and viral genomes. Finally, human TLR11 has been reported to be non-functional because of the presence of a stop codon in the gene and TLR10 is able to homodimerize or heterodimerize with TLR1 and TLR2, but its ligand remains unknown [11].

Recognition of PAMPs by host TLRs triggers intracellular signaling cascades through a set of toll/interleukin-1 receptor (TIR) -domain-containing adaptors, including myeloid differentiation primary response protein 88 (MyD88), TIR domain-containing adaptor protein (TIRAP/Mal), TIR domain-containing adaptor inducing interferon (IFN) (TRIF/TICAM1) and TRIF-related adaptor molecule (TRAM/TICAM2) [12]. Each TLR recruits a specific combination of adaptors to activate different transcription factors, giving rise to appropriate inflammatory responses. All TLRs, with the exception of TLR3, share the adaptor MyD88 which recruits IL-1 receptor-associated kinase-4 (IRAK-4) and leads to its phosphorylation. The phosphorylated IRAK-4 then induces the phosphorylation of IRAK-1 [13], which, in turn, activates the tumor necrosis factor receptor-associated factor 6 (TRAF6). TRAF6 induces phosphorylation of the IκB kinase (IKK) complex, resulting in activation of the transcription factor NF-κB [14].

TIRAP mediates the activation of the MyD88-dependent pathway downstream of TLR2 and TLR4 [15]. TRIF is another adaptor molecule for TLR3 and TLR4 that produces a MyD88-independent [16,17] signaling pathway (TRIF-dependent). This pathway activates both NF-κB and interferon regulatory factor 3 (IRF3) to induce the activation of type I interferons (IFNs), and also causes delayed NF-κB activation mediated through the receptor-interacting protein-1 (RIP1) [17]. TRAM selectively mediates the TRIF-dependent pathway downstream of TLR4, but not TLR3.

### 1.3. Flavonoids

Flavonoids are vegetal compounds that contribute to the brilliant shades of blue, scarlet and orange colors in leaves, flowers and fruits of plants. In addition, they are found in seeds, nuts, grains and spices, and in some beverages such as wine, tea and beer [18]. Chemically, flavonoids are polyphenols which are commonly found conjugated to sugars (as a glycosylated form) although some of them can exist as free aglycones [19].

**Figure 1:**
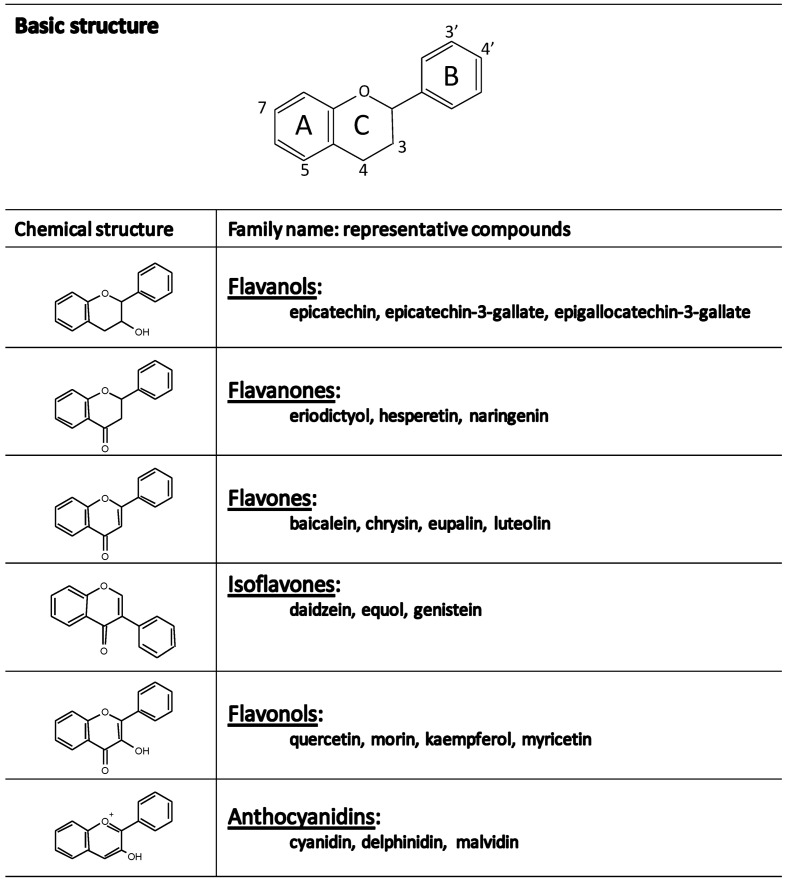
Chemical structure and representative compounds from the main families of flavonoids.

The basic flavonoid structure is the flavan nucleus consisting of 15-carbon skeleton arranged in two phenyl rings bound by a three-carbon bridge commonly cyclized with oxygen, then forming three rings labeled A, B, C (Figure 1) [18]. The various classes of flavonoids differ in the level of oxidation and pattern of substitution on the C ring while individual compounds within a class differ in the pattern on substitution of the A and B rings. The major classes of flavonoids are flavonols, flavones, flavanones, isoflavones, flavanols and anthocyanidins (Figure 1).

Flavonols are ubiquitous in plants and are found mainly in fruit skin, being the yellow and red onions one of the richest sources of them [20]. The flavonol class includes kaempferol, quercetin, and myricetin typically found as glycosides. The flavones, such as apigenin, luteolin, and baicalin, are less widely distributed, although substantial amounts of apigenin and luteolin have been detected in cereal grain and some aromatic herbs (parsley, rosemary, thyme) [18,20]. The most abundant *flavanone* is naringenin, followed by hesperidin, eriodictyol and hesperetin. They are present in especially high amounts in citrus fruits and usually occur as rutinosides and neohesperidosides, which differ in taste [19]. Isoflavones are found most often in leguminous plants with substantial quantities of daidzein and genistein occurring in soybean [20]. *Flavanols* (flavan-3-ols) include monomeric and polymeric forms called procyanidins (condensed tannins). Monomeric forms are catechins and gallic acid esters of catechins and epicatechins [19]. They are widely distributed in plants and fruits and also in tea leaves [18]. *Anthocyanidins* have a fully aromatized C ring and as a result they are positively charged. The most common of these natural water-soluble compounds are pelargonidin, cyaniding, delphinidin, and malvidin, which form conjugates with sugars and organic acids to generate a multitude of anthocyanins that confer a wide range of colors, ranging from orange and red to blue and purple, on fruits (specially in berries like strawberries, blueberries, blackberries and blackcurrants), vegetables and plants [21,22].

## 2. Objective

There is increasing scientific evidence of a relationship between diets rich in natural antioxidants and the prevention of several chronic diseases. The inverse association between dietary antioxidants and inflammatory-like disorders is supported by several epidemiological and interventional studies [23]. Polyphenols, including flavonoids, are the most commonly consumed dietary antioxidants and therefore they may be one of the major responsible for the healthy effect of fruit-and-vegetable-enriched diets. As the activation of the TLR pathway constitutes the first step in the inflammatory cascade activation, and its deregulation can lead to severe chronic inflammation and immune disorders, it is plausible to hypothesize a downmodulatory action of flavonoids in the TLR-induced pathways.

**Figure 2:**
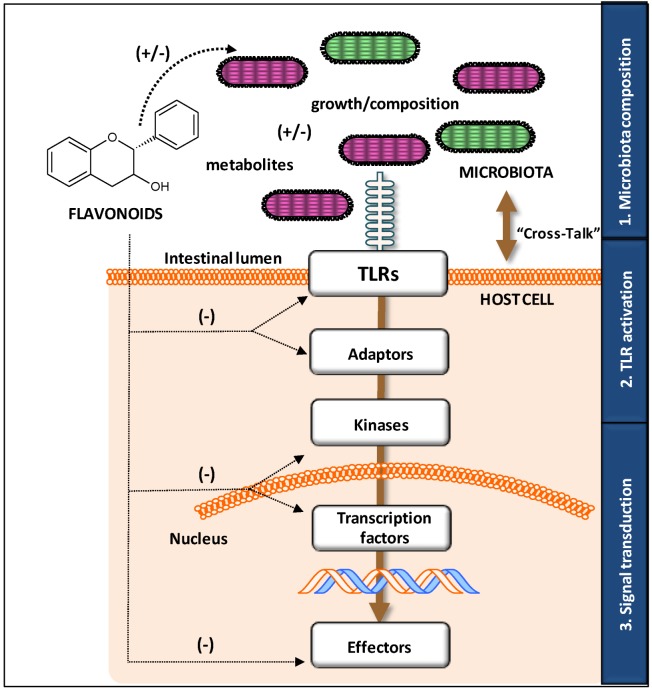
Overview of the mechanisms involved in the regulation of microbiota-host crosstalk by flavonoids. They can act at three different levels by modulating: (**1**) microbiota composition, by means of directly (flavonoid) or indirectly (metabolite) affecting the growth; (**2**) Toll-like receptor (TLR) activation, by means of acting on the receptor and its adaptor proteins; (**3**) signal transduction, by means of interfering with upstream and downstream kinases as well as the transcription factors involved in the inflammatory and immune response activation.

This review focuses on the molecular targets and mechanisms involved in the modulatory action of flavonoids on TLR-mediated signaling pathways. In this context, it aims to provide an overview of the three major levels involved in such action: by varying the composition of the microbiota, by modulating the expression and activation of TLRs and, finally, by modifying the downstream signaling pathways involved. The synergy of all these mechanisms may explain the high potential of flavonoids for preventing certain types of disorders (Figure 2). The suppression of TLR activation by flavonoids offers new potential interventions for ameliorating inflammatory diseases and therefore constitutes a new alternative to current approaches.

## 3. Mechanisms of Modifying “Cross-Talk” by Flavonoids

### 3.1. Influence on Growth and Composition of Microbiota

Bacterial composition (*i.e.*, types of MAMP and PAMP) is one of the major factors influencing TLR activation. In turn, dietary habits are known to modulate the composition of the gut microbiota [24]. For this reason, the earliest level involved in modifying the host-bacteria crosstalk by dietary flavonoids involves the modification of the microbial inhabitants in the gut (Figure 2). There is evidence showing that several kinds of flavonoids, including flavanones, flavonols, flavanols, and isoflavones, modify the microbiota composition. This has been recently reviewed by Etxeberria *et al.* [22] who extensively compile the latest investigations regarding the impact of phenolic compounds, as pure chemicals or in the food context, on microbial communities in both *in vitro* and *in vivo* studies (in animal and human interventions).

With regard to pure flavonoids, an *in vitro* study tested the impact of particular flavonols (rutin, quercetin), flavanols (catechin) and flavanones (naringin, naringenin, hesperidin and hesperetin) on the growth of pure cultures of six bacteria species [25]. The author showed the dose-dependent inhibitory effect of the aglycones naringenin and quercetin on the growth of all bacteria species studied, which was stronger than that of their glycosides [25]. On the other hand, both quercetin (aglycone) and rutin (glycoside) significantly increased the growth of the major intestinal *Bacteroidetes* and *Firmicutes* phyla, with a greater increase in the former than in the latter *in vitro* [26]. However hesperetin and catechin (both aglycones) showed no or weak antibacterial activity against most of the analysed bacteria [25], which was later corroborated by an *in vitro* study evaluating the antimicrobial activity of five aglycones on the growth of the probiotic *Bifidobacterium adolescentis* [27]. Similarly, the repressing and/or stimulating effect induced by *flavanols*, such as catechin and epicatechin, on the growth of certain bacteria has been reported in *in vitro* and batch cultures studies [28,29].

**Figure 3:**
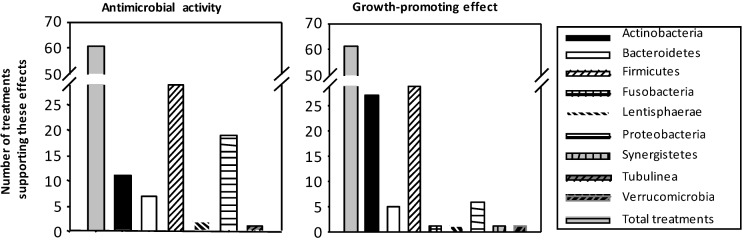
Summary of the number of treatments using beverages (cocoa, tea and wine), fruits or vegetables (soy, pomegranate, grapes, berries and apples) rich in flavonoids and their impact on bacterial growth. There is a high number of studies with flavonoids (*in vitro* and *in vivo*) showing both antimicrobial activity (**left**) and growth-promoting effects (**right**) on the more prevalent bacterial groups.

Besides those studies of particular flavonoids on bacteria *in vitro*, there are a number of studies showing the changes induced by flavonoid-rich foods on microbiota composition. In order to synthesize the available data, and based on the recompilation performed by Etxeberria *et al.* [22], the Figure 3 below classifies the number of studies or treatments with polyphenols, mainly flavonoids, into the antimicrobial activity or the growth-promoting effect on the more prevalent bacterial groups.

Globally, more than 100 experiments have shown that bacterial groups in gut microbiota are affected by beverages (cocoa, tea and wine) and fruits/vegetables rich in flavonoids, either showing antimicrobial activity or, on the contrary, promoting the growth of certain bacteria. These effects have been shown in all types of flavonoids.

Concerning the effect of flavonoid-rich beverages, the intake of cocoa, which mainly contains epicatechin and catechin, and their polymeric forms, was able to inhibit the growth of *Clostridium histolyticum/C. perfringens, Staphylococcus* and *Streptococcus* genera in preclinical and clinical studies [28,30]. In the same way, tea flavonoids exerted an antimicrobial activity against selective bacterial species [31], whereas bifidogenic properties have also been attributed to them [32,33]. Differences in the proportion of the four major bacteria phyla in humans (*Proteobacteria*, *Fusobacteria*, *Firmicutes*, and *Bacteroidetes*) have been observed after a nutritional intervention with red wine which contains a complex mixture of polyphenols, mainly including flavanols and anthocyanins [34]. Similar effects have been described in rats treated for 16 weeks with wine polyphenol, which showed a predominant proportion of *Lactobacillus* and *Bifidobacterium* spp. [35], while controversial results were obtained in an *in vitro* batch culture [33,36]. Nevertheless, these effects were not so pronounced or even disappeared in humans when consuming dealcoholized red wine [34].

Changes have also been observed in the *Firmicutes*, *Actinobacteria* and/or *Bacteroidetes* proportions induced by soy products, which mainly contain isoflavones, in *in vitro*, preclinical and clinical studies [37,38,39,40]. In addition, the potential modulation of gut microbiota composition by fruits has been thoroughly reviewed by Etxeberria *et al.* [22]. To date, a large amount of evidence suggests that fruits and their derivatives are able to significantly boost the growth of colonic-friendly bacteria, such as *Bifidobacterium* and *Lactobacillus* [41,42,43,44].

Overall, it can be summarized that the intake of flavonoids is able to modulate the composition of the gut microbiota, potentiating the growth of specific beneficial bacteria strains (*Lactobacillus and Bifidobacterium* in >70% of the studies), and also inhibiting the growth of certain pathogenic bacteria (*i.e., Clostridium*). It must be added that, despite the evidence described above, natural flavonoids are not the only responsible for this effect on gut microbiota but their metabolites are also involved. Flavonoids and related compounds can reach the colon where they become substrate for the microbial metabolism [45,46]. Indeed, intestinal bacteria have the ability to metabolize flavonoids, more or less efficiently, depending on their chemical structure, to simpler and more bioavailable metabolites [20,47]. This transformation modulates the biological activities of these compounds, which, in general, are more beneficial after colonic metabolism than in their original forms [45,46,48,49,50]. Besides the known biological activity of most small phenolic metabolites, several microbial catabolites from epicatechin, epigallocatechin or naringenin have been described possessing important antimicrobial activity, especially against Gram-negative species [51].

### 3.2. Modulatory Action on TLR Gene and Protein Expression

TLR pathways can be modulated by flavonoids at different levels (Figure 2). Both TLR gene expression and cell membrane expression, which are directly related with TLR functionality, can be affected by flavonoids. Under homeostatic conditions IEC have low expression of TLR2 and TLR4, and therefore in a healthy context, TLR activation is low; however, in an inflammatory scenario, TLR expression on IEC increases and then TLR signaling is triggered [52].

Both *in vitro* studies (Table 1), using primary cells or cell lines, and *in vivo* studies (Table 2) have evidenced the influence of different classes of flavonoids on TLR gene and protein expression. The best results regarding the down-regulatory effect of flavonoids have been raised in the presence of TLR activators (*i.e.*, LPS as an *in vitro* TLR4 activator) whereas lack of effect is found under non stimulatory conditions (*i.e.*, in healthy animals/subjects).

LPS-stimulated J774 macrophages treated with the flavonols engeletin and astilbin showed inhibition of TLR4 gene expression [53]. In addition, kaempferol-3-*O*-sophoroside down-regulated the cell surface expression of TLR2 and TLR4 in endothelial cells [54] as did quercetin in PBMC after challenge [55]. Flavanones as naringenin and hesperidin have shown similar effects. In particular, naringenin down-regulates TLR2 and TLR4 protein expression and TLR2 mRNA levels in PBMC after being challenged with *Chlamyidia trachomatis* [56]. Other assays performed *in vitro* in 3T3-L1 cells during adipocyte differentiation and *in vivo* in C57Bl/6j mice after a high fat diet (HFD) agree with the inhibitory effect of naringenin on TLR2 gene expression, although no effect was found for TLR4 [57].

**Table 1:** Flavonoids on TLR gene and protein expression in *in vitro* studies.

Flavonoid	Dose	Duration ^1^	TLR ^2^	Studied Target	Cell (Challenge)	Reference
**FLAVONOLS Engeletin**	10/50 μM	2 h	↓ TLR4	mRNA	J774 macrophages (LPS)	[53]
**Astilbin**			↓ TLR4	mRNA		
**Kaempferol-3-*O*-sophoroside**	–	6 h	↓ TLR2	Protein	Endothelial cells	[54]
			↓ TLR4			
**Quercetin**	25 μM	1 h	↓ TLR2	Protein	PBMC	[55]
			↓ TLR4			
**FLAVANONES Naringenin**	1 μg/mL	48 h	↓ TLR2	Protein/mRNA	J774 macrophages (*C. trachomatis*)	[56]
			↓ TLR4	mRNA		
**Naringenin**	100 μM	–	↓ TLR2	mRNA	3T3L1 cells (adipocyte differentiation)	[57]
**FLAVANOLS Epigallocatechi*n*-3-gallate**	1 μM	24 h	↓ TLR4	Protein/mRNA	RAW 264.7 macrophages (LPS)	[58]
**Epigallocatechi*n*-3-gallate**	10 μM	24 h	↔ TLR4	mRNA	Murine bone marrow-derived DCs (unstimulated)	[59]
**FLAVONES Baicalin**	40/80 μM	24 + 3 h	↔ TLR1-9	mRNA	Human oral keraynocytes (LPS)	[60]
**Baicalin**	5/10 μg/mL	0.5–6 h	↓ TLR2	Protein/mRNA	PC12 and primary rat neurons (oxygen glucose deprivation)	[61]
			↓ TLR4	Protein/mRNA		

Notes: ^1^ Treatment duration is showed when detailed in the article, if it is not provided a (–) is showed; ^2^ Changes in gene expression are summarized by means of an increase (↑), decrease (↓) or not affected (↔) by the intervention.

**Table 2:** Flavonoids on TLR gene or protein expression in *in vivo* studies.

Flavonoid	Dose	Duration	TLR ^1,2^	Studied Target	Cell	Reference
FLAVONES Luteolin	Daily dose of 10 and 25 mg/kg body weight	24/78 h	↓ TLR4	Protein/mRNA	Cerebral cortex from. p.o ^3^ fed SD rats	[62]
			↓ TLR5			
Baicalin	One dose of 50 mg/kg body weight	4 h	↓ TLR2	Protein/	Mice hippocampus cells (carotid arteries ligation)	[61]
			↓ TLR4	mRNA		
				mRNA		
FLAVANONES Naringenin	1% included in food	16 weeks	↓ TLR2	mRNA	Adipocytes C57Bl/6J mice after HFD	[57]
EXTRACTS *Achyrocline satureoides* (Quercetin and Luteolin)	One dose of 100 mg/kg	1 h	↓ TLR4	Protein	Neutrophils from p.o fed Wistar rats	[63]
Cocoa (Procyanidins)	5%/10% included in food	3 weeks	↔ , ↓ TLR2	mRNA	Small intestine and mesenteric lymph nodes from p.o fed Wistar rats	[64]
			↔ , ↔ TLR4			
			↔ ,↓ TLR7			
			↔ , ↑ TLR9			
Cocoa (Procyanidins)	10%included in food	7 weeks	↔ , ↓ , ↔ TLR2	mRNA	Small intestine, Peyer’s patches and mesenteric lymph nodes from p.o fed Wistar rats	[65]
			↓ , ↑, ↔ TLR4			
			↔ , ↓ , ↔ TLR7			
			↔ , ↑ , ↑ TLR9			
Cocoa (Procyanidins)	10% included in food	6 weeks	↔ TLR2	mRNA	Large intestine from p.o fed Wistar rats	[30]
			↔ TLR4			
			↔ TLR7			
			↓ TLR9			
Orange juice (Flavanones)	One dose of 300 kcal drink of orange juice	1/3/5 h	↓ TLR2	mRNA	Mononuclear cells from healthy subjects given high-fat high-carbohydrate meal	[66]
			↓ TLR4	Protein		
Orange juice (Flavanones)	One dose of 300 kcal drink of orange juice	1/3/5 h	↔ TLR4	mRNA	Mononuclear cells from healthy subjects given high-fat high-carbohydrate meal	[67]
				Protein		

Notes: ^1^ The TLR changes are expressed as the effect on each studied tissue in the same order as mentioned on the cell/challenge column separated among them with a comma; ^2^ Changes in gene expression are summarized by means of an increase (↑), decrease (↓) or not affected (↔) by the intervention; ^3^ p.o. means *per os* or administration by oral route.

Flavones have been shown to also modulate TLR gene expression in both *in vitro* and *in vivo* studies. Isoliquiritigenin decreased, among other targets, the gene expression of TLR4 in RAW 264.7 macrophage line [68], and luteolin administrated at different doses to rats was able to suppress the overexpression of TLR4 and TLR5 induced by ischemia in the cerebral cortex at both protein and gene level [62]. Baicalin added to human oral keratynocytes culture was able to down-regulate *P. gingivalis* LPS-induced expression of downstream genes associated with TLR signaling, but almost no changes were found in the gene expression of TLR1-9 determined by the PCR array [60]. However, in another study, baicalin down-regulated protein and gene expression of TLR2 and TLR4 both *in vitro*—PC12 and primary neurons under oxygen glucose deprivation conditions—and *in vivo*—hippocampus cells from mice after carotid arteries ligation [61].

With regard to flavanols, gene and cell surface expression and total protein level of TLR4 were markedly reduced by the addition of epigallocatechin gallate (EGCG) to LPS-stimulated RAW 264.7 macrophages [58]. However, it did not affect TLR4 gene expression when murine bone marrow-derived DCs were in resting conditions [58], which is in agreement with other studies where the flavonoid is not able to modify TLRs in the absence of stimulatory conditions. On the contrary, a semi-synthetic flavonoid called flavopiridol did not alter the TLR4 cell surface expression in LPS-stimulated RAW 264.7 macrophages [69].

Some extracts rich in flavonoids have also shown an effect on TLR gene expression, mainly in *in vivo* studies. Thus, the oral treatment of Wistar rats with a hydroalcoholic extract of *Achyrocline satureoides*, a rich source of quercetin and luteolin, decreased TLR4 expression on neutrophils [63]. Wistar rats fed cocoa, a rich source of procyanidins among other flavonoids, showed changes in the expression of several TLR in different tissues of the intestinal immune system [64]: a cocoa diet induced an up-regulation of TLR4 and TLR9 gene expression and a down-regulation of TLR2 and TLR7 gene expression in Peyer’s patches (PPs) and mesenteric lymph nodes (MLNs). Conversely, in small intestine (SI) tissue, cocoa-fed animals showed lower values for TLR4 and TLR9 mRNA and a higher value for TLR2 and TLR7 [65]. However, a similar design did not show a clear effect on colonic tissue, where microbiota is mainly found [30]. It should be taken into account that the effects induced by these extracts cannot be exclusively attributed to flavonoids because other components may be acting as well (*i.e.*, theobromine and fiber in cocoa).

In humans, two interventional studies using orange juice have evaluated the increase in TLR2 and TLR4 mRNA and protein expression in PBMC, induced by a high-fat high-carbohydrate meal, in volunteers [66,67].

In addition to flavonoids, the effect on TLR genes or surface expression by other polyphenols has also been reported. Thus, curcumin decreases TLR2 gene and surface expression in immune cell lines (monocytic THP-1 cells and dHL-60 promyelocytic leukaemia cells) and in primary cells (peripheral blood polymorphonuclear neutrophils, PMN) [70], and a complement containing resveratrol prevented the increase in TLR4 gene expression in PBMC induced by a high-fat, high-carbohydrate meal in healthy subjects [71].

Finally, it has recently been described that TLR gene expression is subject to circadian rhythms [72,73] which might be related to the presence of flavonoids and other exogenous regulators of TLR in the diet.

It should be also mentioned that certain flavonoids *in vivo* may not be the only direct responsible of the induced effects because indirect effects, through one or more of its microbial metabolites, might be also considered. However, in the *in vitro* studies the TLR modulation should be due to direct effects because the microbial metabolism is not present in such approach. Further studies with this aim should be developed.

### 3.3. Modulatory Action in TLR Activation

The activation of TLR intracellular signaling activation requires further processes that can also be targeted by flavonoids. These processes include the accumulation of lipid rafts, microdomains rich in cholesterol and sphingolipids that act as platforms for downstream molecules and seem to be a key factor in the activation of immune cells, TLR dimerization/oligomerization [74], its glycosilation and the participation of adaptor proteins.

Thus, quercetin and luteolin could interact with lipid rafts [75], whereas isoliquiritigenin was able to suppress LPS-induced dimerization of TLR4 in a dose-dependent manner in RAW 264.7 macrophage cell line [68]. Polyphenols such as curcumin and resveratrol have also shown this effect [74]. With regard to the influence on TLR glycosilation, *Helicobacter pylori* infection seems to initiate the inflammatory cascade in gastric epithelial cells by TLR4 glycosilation, fact that has been reported suppressed by EGCG [47].

**Figure 4:**
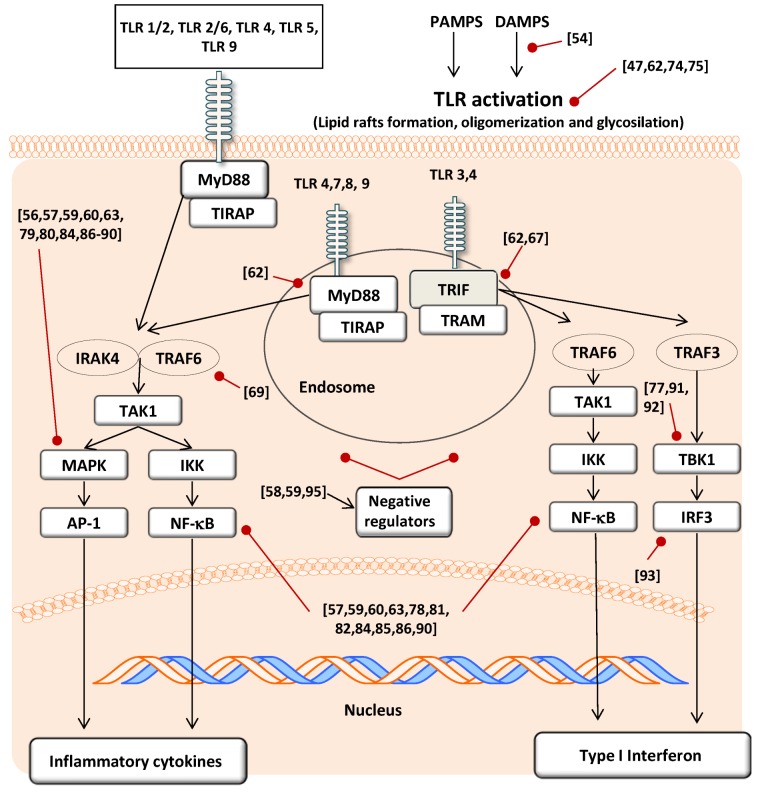
Signal transduction molecules affected by the flavonoids modulatory action on TLR activation. Both, the MyD88 dependent (**left**) and independent (**right**) pathways are modulated by the effect of several flavonoids on targets from different upstream (TLR expression and activation; adaptors modulation) and downstream levels (kinases and transcription factors). Black arrows indicate stimulation of the pathway and red arrows indicate inhibition of the pathway. In brackets there are the references cited in the text.

There are at least five adaptor proteins of TLR and each of these has a TIR domain (*i.e.,* TRIF or MyD88), which directly or indirectly binds to the TIR domain of a TLR [76]. Flavonoids seem to modulate the pathways involving such adaptors, however from the literature it is difficult to ascertain which is the specific target affected and mainly focus on downstream molecules (Figure 4). As examples, isoliquiritigenin in RAW 264.7 macrophages was able to suppress the MyD88-dependent pathway (*i.e.*, IRAK1 degradation) [68] whereas other *in vitro* studies performed in RAW 264.7 cells and also in 293T (human embryonic kidney cell line) showed that EGCG treatment was able to modulate both MyD88- and TRIF-dependent signaling pathways [77].

On the other hand, it has to be taken into account that TLR gene expression can be self-regulated by downstream molecules participating in its signaling (*i.e.*, NF-κB) [78], which potentiates the down-modulatory effect of those flavonoids able to act on both targets.

### 3.4. Effect on Signal Transduction Molecules

A large number of articles can be found regarding the effect of flavonoids on the activity of different protein kinases that regulate various intracellular signaling cascades, such as MAPK [79,80]. For this reason flavonoids may also modify the downstream kinases activated after TLR triggering. Furthermore, oxidative stress accompanying inflammation, which also leads to MAPK activation, seems to be controlled by the direct action of flavonoids (Figure 4).

The activation of MAPK engages ERK 1 and 2, JNK and p38 MAPK linked to stress stimuli, such as ROS overproduction or inflammation, and among other actions, this stimulation induces the phosphorylation of inhibitor proteins such as κB inhibitor proteins (IκBs), involved in the NF-κB pathway. The modulation of the activity of NF-κB, a pro-inflammatory transcription factor, is one of the clearest effects of certain flavonoids [81,82]. NF-κB triggers the expression of over 100 genes, many of them involved in the inflammatory response [83]. NF-κB is found in the cytoplasm of non-stimulated cells bound to IκBs which are modulated by MAPK action.

To date in the TLR context, the flavanols epicatechin gallate (ECG) and EGCG in human dental pulp fibroblast were able to block the ligand-stimulated TLR2 cell signaling pathways throughout the down-regulation of downstream mechanisms such as p38MAPK, SAP/JNK, ERK1/2 and phosphorylation of IkBa and p65 subunit of NF-κB [84]. Likewise, in DC, EGCG inhibited LPS-induced MAPK and NF-κB activation [59].

Flavones, flavonols and flavanones also modulate these kinases. Luteolin inhibits p38 MAPK [62] and IκB kinase activity [85] both indicating a role for this flavonoid in MyD88-dependent signaling pathway. Baicalin may inhibit *P. gingivalis* LPS-induced activation of NF-κB, p38 MAPK and JNK [60], and same inflammatory pathway when the study was performed in isquemic neurons [86]. The flavonol flavopiridol—quercetin analogue—was also able to regulate the downstream TLR2 and TLR3 pathways through the MyD88-dependent pathway but not the independent pathway, as was demonstrated by *in vitro* studies showing inhibition of the activation of NF-κB and MAPKs but not TRAF6 [69]. Flavanones such as naringenin inhibited TLR2 pathways by the modulation of IκB degradation and JNK phosphorylation [57], and also down-regulated the induced increase of phosporylation of p38 MAPK in macrophages infected with *Chlamyidia trachomatis* [56].

Foods rich in flavonoids, such as cocoa, have also shown this potential *in vitro*, either by their scavenging action or by direct interaction with the downstream kinases [87,88]. In this context, epicatechin, catechin and dimeric B procyanidins seem to be accumulated in the cytosol and act at the early stages of NF-κB activation, regulating oxidant levels and reducing IκBs phosphorylation, whereas cocoa dimeric procyanidins seem to be able to enter the nuclei and selectively avoid NF-κB binding to DNA [89].

Overall, it is plausible that the different structures of flavonoids and their regiospecific modifications in their side chains lead to different bioactivities. In this sense, Lim *et al.* [90] studied the effect of five classes of plant-derived flavonoids (flavonols, flavones, flavanones, catechins and cyanidins) on TLR2 pathway *in vitro* and found differential effects on the targets studied, such as p38 MAPK and NF-κB.

TBK1, a representative kinase found in TRIF-dependent TLR activation, participates in IRF3 activation and type I IFNs production (Figure 4). Some flavonoids, such as EGCG, luteolin, quercetin, chrysiin and eriodictyol, showed inhibitory effects on this downstream kinase whereas others, such as naringenin and hesperitin, had no effect [77,91,92]. Moreover, isoliquiritigenin was able to suppress TLR3 and TLR4 agonist-induced IRF3 activations in RAW 264.7 macrophage cell line [93]. These results demonstrated the down-regulatory activity of certain flavonoids on the TLR3 and TLR4 TRIF-dependent pathways (Figure 4).

Another mechanism can be proposed as an alternative to the direct actions of flavonoids on specific targets of the TLR pathway. In recent years, the importance of the intracellular negative regulators of TLRs such as SOCS1, TOLLIP, IRAK or SIGIRR has been described [94]. In this regard, procyanidin dimer B2-treated macrophages up-regulate IRAK-M protein and therefore also suppress TLR4 signaling and phosphorylation of MAPKs such as ERK1/2, p38 and JNK [95]. Moreover, EGCG has been shown to up-regulate TOLLIP protein expression through a 67-kDa Laminin receptor (67LR) in macrophages [58] and in DCs [59].

Furthermore, as previously described, DAMPs can also activate TLR pathways and it seems that flavonoids can target these molecules as well. For example, a DAMP known as high mobility group box protein 1 (HMGB1) acts as an endogen ligand of TLR2 and TLR4 when it is released to the extracellular compartment [6] and this release by endothelial cells is modulated by kaempferol-3-*O*-sophoroside [54].

Overall, it can be suggested that changes in microbiota composition and TLR modulation by flavonoids may have also an impact on the intestinal immune activation and oral tolerance. In this sense, flavonoids by modulating intestinal microbiota (Section 3.1) may polarize the immune response towards the T-cell mediated tolerogenic pathway. At the same time the repression of the TLR signaling, which is associated to innate immune tolerance [96], can be also produced by flavonoids (Section 3.2). As certain flavonoids have been described to present immunomodulator activity by inducing changes on the GALT [97] and on systemic immune cells [98], as well as on regulatory T cells [99,100], we may suggest the possible implication of TLR signaling modulation herein described by flavonoids in such immunoregulatory effects.

## 4. Conclusions

Flavonoids are able to act as exogenous regulators of TLR and therefore modulate the host-microbiota crosstalk. This is possible due to their ability to modulate the TLR signaling pathways at different levels. On one hand, flavonoids can modify the composition of the microbiota, either by direct action or through their microbial catabolites, acting as antimicrobial agents on certain bacterial groups or as growth promoters for other bacteria. On the other hand, flavonoids can modulate the gene expression, the initial activation process of the receptor, and the upstream and downstream signaling molecules involved in the TLR pathway. These synergistic mechanisms make some flavonoids interesting modulatory agents on TLR-mediated inflammatory responses and therefore it is plausible to think that their preventive effect on certain chronic diseases, associated with exacerbated TLR activation, may be at least in part result from TLR regulation.

Based on these effects, we suggest that more preclinical, *in vitro* and animal model, studies, as well as human clinical trials, should be carried out with flavonoids regarding this aspect. Taking into account the lack of toxic effects and the high availability from natural sources, they should be considered as tools to counteract the increase in inflammatory-based diseases in our society.
